# Anterior vertebrectomy and O-arm navigation for old L5 traumatic fractures with kyphotic deformity: a retrospective case series of clinical and radiological outcomes

**DOI:** 10.3389/fsurg.2026.1750755

**Published:** 2026-04-29

**Authors:** Zhi-da Chen, Yuan-jie Jiang, Bin Lin, Xiao-yang Hu, Yu-zhe Zeng, Hui Liu, Tao-yi Cai

**Affiliations:** Department of Orthopaedics, the 909th Hospital, School of Medicine, Xiamen University, Zhangzhou, China

**Keywords:** anterior vertebrectomy, kyphotic deformity, o-arm navigation, old l5 traumatic fractures, spinal fusion, surgery

## Abstract

**Background:**

Old L5 traumatic fractures with kyphotic deformity are quite rare and surgical management constitutes a significant challenge. Anterior vertebrectomy and reconstruction with O-arm navigation for old L5 traumatic fractures with kyphotic deformity aim to restore spinal stability, correct deformity, and improve functionality. This study evaluates the efficacy and safety of this approach.

**Methods:**

A retrospective case series was conducted on 43 patients with old L5 traumatic fractures and kyphotic deformity who underwent anterior vertebrectomy and reconstruction using O-arm navigation. Data were collected on operation duration, blood loss, radiological and clinical outcomes (VAS, ODI, ASIA, local Cobb angle, and vertebral anterior margin height ratio), Bridwell classification, and complications.

**Results:**

The mean operation duration was 182.5 ± 32.8 min, with an average blood loss of 570.5 ± 71.4 mL. All patients had regular follow up with an average duration of 27.1 ± 6.8 months. The VAS scores and ODI at 3 months postoperatively and at the final follow-up showed significant improvement compared to preoperative scores (*P* < 0.05). VAMHR improved significantly from 35.9 ± 5.6% preoperatively to 92.1 ± 2.1% at the final follow-up (*P* < 0.001). The LCA at the final follow-up 14.4 ± 3.7° showed statistically significant difference compared to preoperative measurements 37.8 ± 2.4°. Preoperative ASIA grades were C in 1 patient, D in 18 patients, and E in 24 patients. And ASIA grades were D in 9 patients and E in 34 patients at the final follow-up. Wilcoxon signed-rank test showed a significant improvement in ASIA grade at the final follow-up compared with preoperative status (*P* < 0.001). 11 of 43 patients (25.6%) improved by one grade, 32 of 43 patients (74.4%) remained unchanged. 34 patients achieving Bridwell grade I bone fusion. Complications were minimal, with 1 case of intraoperative venous bleeding successfully managed.

**Conclusion:**

Anterior vertebrectomy and reconstruction using O-arm navigation is an effective and safe approach for treating old L5 traumatic fractures with kyphotic deformity in the medium term follow-up. It offers significant pain relief, functional recovery, and sustained correction of spinal alignment, with low complication rates and high fusion success.

## 1 Introduction

Isolated L5 traumatic fractures are rare, accounting for approximately 1.2% of all spinal injuries (1, 2). These fractures typically result from high-energy trauma, such as falls or motor vehicle accidents (3). The L5 vertebra plays a critical role in the lumbosacral junction, bearing substantial biomechanical loads and acting as a transition point between the mobile lumbar spine and the rigid pelvis. This unique anatomical position, combined with the orientation of facet joints and the sacral slope, makes L5 particularly susceptible to axial compressive forces and shear stress during injury. The injury mechanism often involves excessive axial loading combined with flexion or rotation, leading to vertebral height loss, retropulsion of bony fragments, and potential spinal canal compromise (4). Over time, untreated or inadequately managed L5 traumatic fractures may progress to kyphotic deformities and neurological complications (5).

Anterior vertebrectomy combined with spinal reconstruction has emerged as a viable option for addressing severe kyphotic deformities and instability following old L5 traumatic fractures. Anterior approaches offer superior access for decompression and correction of deformity compared to posterior techniques (6). However, these methods come with risks such as vascular injury and increased operative complexity, necessitating adjunctive tools to enhance precision and safety. Among these, O-arm navigation technology has gained prominence, providing real-time three-dimensional imaging that facilitates precise screw placement and alignment (7, 8). This technology not only enhances intraoperative visualization, but also minimizes the risks associated with conventional fluoroscopy-guided techniques (9).

Despite advancements in surgical techniques and navigation tools, the management of old L5 traumatic fractures with kyphotic deformity remains a contentious topic. While some studies advocate conservative management for stable fractures, surgical intervention is often necessary for patients with instability, neurological compromise, or progressive deformity (1, 4, 10). This study aims to evaluate the clinical and radiological outcomes of anterior vertebrectomy and reconstruction using O-arm navigation for old L5 traumatic fractures with kyphotic deformity, providing insights into the safety, efficacy, and benefits of this approach.

## 2 Methods

### 2.1 Patient selection

This retrospective study analyzed 43 patients who underwent anterior vertebrectomy and reconstruction with O-arm navigation for old L5 traumatic fractures accompanied by kyphotic deformity from January 2018 to December 2023. Preoperatively, all patients underwent lumbar spine x-rays, 3D-Computed Tomography (3D-CT), and magnetic resonance imaging (MRI) to determine the presence of vertebral body fractures, spinal cord compression, and kyphotic deformities. The surgeries were performed at a single institution, and data collection spanned from initial surgery to final follow-up. All procedures were carried out by experienced spinal surgeons. Ethical approval was obtained, and all participants provided informed consent.

Patients included in the study had old L5 traumatic fractures with objective radiographic kyphotic deformity and instability. All patients had delayed presentation after initial non-operative management or inadequate early treatment, and patients with osteoporotic fractures were excluded. Significant kyphotic deformity was defined as a local Cobb angle >20°, measured between the superior endplate of L4 and the inferior endplate of S1. Instability was determined using dynamic position radiographs and was defined as abnormal motion at the injured lumbosacral segment, including sagittal translation >3.0 mm or intervertebral angulation change >10° at the injured segment (11). Exclusion criteria included active infections, prior lumbar spine surgeries at the affected level, non-surgical treatment, and severe comorbidities that contraindicated major surgery.

### 2.2 Operative procedure

All patients underwent combined approach surgery using general anaesthesia. According to the LCA, degree of spinal cord compression, and presence of disc herniation, different surgical sequence of procedures, and fixation segments were selected.

Posterior approach: patients were placed in a prone position. A midline incision is made over the lumbar spine and a reference frame for O-arm navigation is placed on the posterior superior iliac spine. Based on the navigation data, the entry point for the pedicle screws is identified at the line connecting the lateral border of the superior articular process and the midline of the transverse process. A hand awl is used to create the screw trajectory, and a probe confirms the bony walls. Pedicle screws are inserted bilaterally under O-arm fluoroscopic guidance. Two titanium rods of appropriate length are pre-bent and installed. On one side, the distal pedicle screws are first tightened to secure the rod. Using a distractor along the rod's direction, the L5 vertebra is repositioned under compression or distraction. The proximal screws are then tightened. The same procedure is repeated on the contralateral side. C-arm fluoroscopy confirms proper alignment and fixation of L5. The surgical site is irrigated with saline, and meticulous hemostasis is performed. A drain is placed, and the layers are closed sequentially.

Anterior approach: patients were positioned to a right lateral decubitus position. A left-sided retroperitoneal approach was used. A slanted 8 cm incision is made along the left mid-axillary line extending toward the anterior axillary line. Surrounding soft tissues are carefully dissected, and the psoas muscle is exposed. Blunt dissection is performed anterior to the psoas muscle to access the L5 vertebra. Preoperative assessment of the vascular corridor was routinely performed. When the vascular space was narrow, assistance from vascular surgery or general surgery was obtained to facilitate safe exposure. A guidewire is inserted, and progressively larger dilators are used to create access. A retractor is placed to expose the L5 vertebral body. When the iliac crest limited the operative field, exposure was optimized by adjustment of patient positioning and retractor trajectory. Iliac crest osteotomy was not routinely required. Under fluoroscopic guidance, the L5 vertebra is identified and prepared. An ultrasonic bone scalpel and rongeurs are used to resect the L5 vertebral body and intervertebral discs at L4-L5 and L5-S1. Retropulsed bone fragments are carefully removed to decompress the spinal canal. Under O-arm fluoroscopic guidance, an expandable cage filled with autologous and allograft bone is inserted between L4 and S1 to restore height and alignment. Fluoroscopy confirms the position of the implant. The surgical site is irrigated with saline, and hemostasis is achieved. A drain is placed, and the layers are closed sequentially. The wound is covered with a sterile dressing.

### 2.3 Postoperative management and observation indicators

All patients received intravenous intravenous antibiotics, pain relief and prevention of deep vein thrombosis. And the patients were encouraged to functional exercise with the assistance of a lumbosacral brace beginning 3 days after surgery. This was continued for an average of 3 months postoperatively.

Operation time (skin-to-skin time), intraoperative blood loss, and occurrence of complications were recorded and patients received regular follow-ups every three months in the hospital's outpatient clinic. Clinical assessments were assessed using the Visual Analogue Scale (VAS: 0 = no pain, 10 = worst imaginable pain), ASIA grading, and the Oswestry Disability Index (ODI). Fusion outcomes were graded using the Bridwell classification system (12), and complications were recorded. Kyphotic deformity was quantified using the local Cobb angle (LCA), formed by the angle between a line drawn parallel to the superior endplate of L4 and a line drawn parallel to the inferior endplate of S1. The vertebral anterior margin height ratio (VAMHR) was accomplished by the ratio of the L5 vertebral anterior margin height to posterior margin height multiplied by 100%. Outcomes were assessed preoperatively, at 1 week and 3 months postoperatively, and at the final follow-up. All patients were followed regularly in the outpatient clinic.

### 2.4 Statistical analysis

Data were analyzed using SPSS 21.0 (SPSS Inc., Chicago, IL, USA). Continuous variables were presented as mean ± standard deviation (SD). For repeated continuous outcomes assessed at four time points (preoperative, 1 week postoperatively, 3 months postoperatively, and final follow-up), one-way repeated-measures analysis of variance (repeated-measures ANOVA) was used. When the overall test was significant, *post-hoc* pairwise comparisons with Bonferroni correction were performed. ASIA grade was analyzed using the Wilcoxon signed-rank test for paired data. The Bridwell classification and complications were analyzed descriptively, A two-sided *P* < 0.05 was considered statistically significant.

## 3 Result

### 3.1 Population characteristics

There were 43 cases of old L5 traumatic fractures with kyphotic deformity, with 27 males and 16 females. Mean age was 39.6 ± 17.2 years (range, 21–52 years). The average operation duration was approximately 182.5 ± 32.8 min (range, 159–211 min). Intraoperative blood loss was measured at an average of 570.5 ± 71.4 mL (range, 450–730 mL). Patients were followed for an average duration of 27.1 ± 6.8 months. Detailed information is shown in Table 1.

**Table 1:** Detail information of the patients.

Variable	Value
Gender	
Male [*n* (%)]	27 (62.8)
Female [*n* (%)]	16 (37.2)
Age [year (range)]	39.6 ± 17.2 (21–52)
Operation duration [min (range)]	182.5 ± 32.8 (159–211)
Intraoperative blood loss [mL (range)]	570.5 ± 71.4 (450–730)
Follow-up [month (range)]	27.1 ± 6.8 (19–32)
Bone union [month (range)]	7.3 ± 3.5 (6–9)
Complication [*n* (%)]	1 (2.33)

### 3.2 Clinical efficacy and complications

Preoperatively, the mean VAS score was 5.6 ± 1.1, which decreased significantly to 3.5 ± 0.5 at 1-week postoperatively (*P* < 0.001). This improvement continued progressively, reaching 2.2 ± 0.6 at 3 months post-surgery (*P* < 0.001 compared to both preoperative and 1-week scores) and further declining to 1.0 ± 0.8 at the final follow-up (*P* < 0.001 compared to all previous time points). Similarly, ODI scores showed a marked decline following the surgery. The preoperative ODI score was 69.9 ± 3.2, significantly improved to 32.0 ± 4.9 one week postoperatively (*P* < 0.001). The improvement was sustained and enhanced over time, with scores reducing to 22.1 ± 5.0 at 3 months (*P* < 0.001 compared to preoperative and one-week scores) and reaching 11.8 ± 3.9 at the final follow-up (*P* < 0.001 compared to all earlier measurements). Preoperative ASIA grading was as follows: 1 case was grade C, 18 cases were grade D, and 24 cases were grade E. At the final follow-up, 9 cases were grade D and 34 cases were grade E. Wilcoxon signed-rank test showed a significant improvement in ASIA grade at the final follow-up compared with preoperative status (*P* < 0.001). 11 patients improved by one grade, 32 patients showed no change, and none showed deterioration.

The surgical procedure demonstrated a low complication rate, with intraoperative venous bleeding occurring in only 1 case (2.33%). This complication was effectively managed through ligation of the iliolumbar vein, ensuring hemostasis without further incident. Importantly, no cases of surgical site infection, nerve injury, or cerebrospinal fluid leakage occurred.

### 3.3 Radiological evaluation

Preoperatively, the mean LCA was 37.8 ± 2.4°, which was significantly reduced to 12.4 ± 5.2° within the first postoperative week (*P* < 0.001). At 3-months post-surgery, the LCA remained stable at 12.2 ± 2.9°, with no statistically significant changes from the 1-week postoperative measurement (*P* > 0.05). However, the improvement remained significant compared to the preoperative value (*P* < 0.001). At the final follow-up, the LCA measured 14.4 ± 3.7°, reflecting a slight increase compared to the 3-month value but remaining significantly improved compared to the preoperative angle (*P* < 0.001). The preoperative VAMHR was (35.9 ± 5.6)% and the 1-week postoperative VAMHR was (93.3 ± 4.9)%, and the 1-week postoperative VAMHR was greater than that of the preoperative period (*P* < 0.001). The VAMHR remained stable at (92.5 ± 2.7)% at 3-months post-surgery and (92.1 ± 2.1)% at the final follow-up, with no statistically significant changes from the 1-week postoperative measurement (*P* > 0.05). Bone union was achieved between 5 and 11 months postoperatively, with an average time of 7.3 ± 3.5 months for bone union. There were 79.1% (34/43) of patients achieving grade I fusion, 20.9% (9/43) of patients achieving grade Ⅱ fusion at the final follow-up.

**Figure 1:**
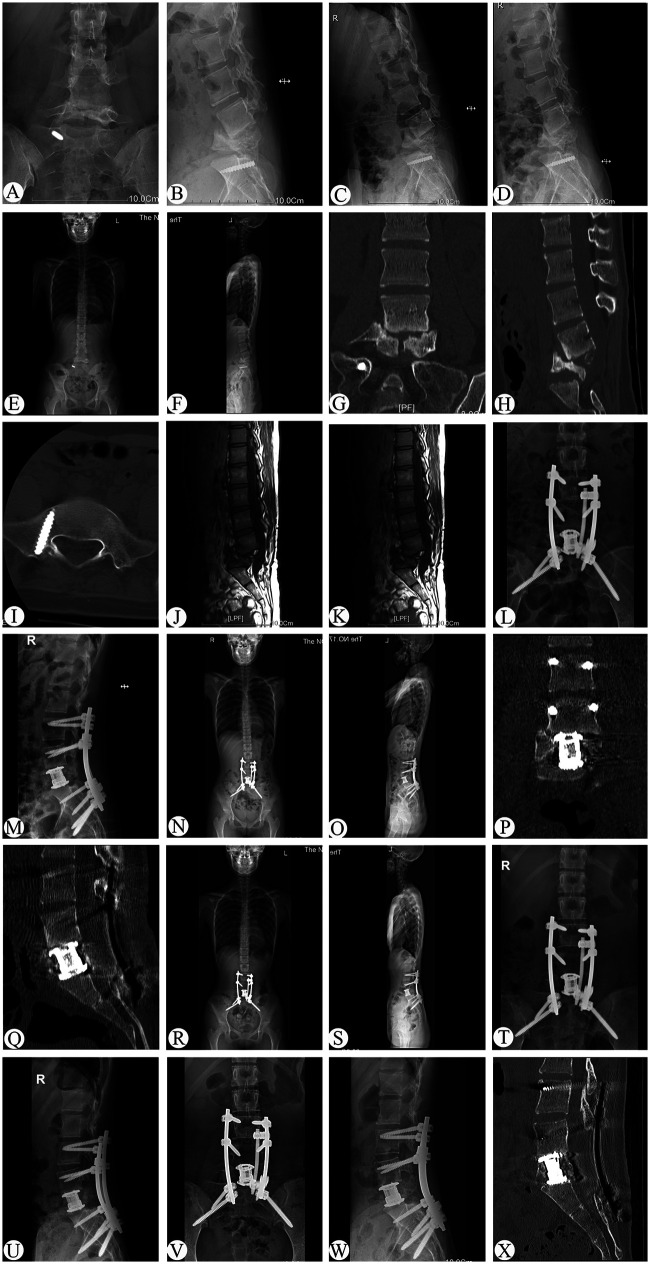
Preoperative, postoperative, and follow-up radiographs of a 49-year-old patient the patient who had dated L5 vertebral fractures with kyphotic deformity managed by anterior vertebrectomy and reconstruction with O-arm navigation (case 8). **(A**-**K)** x-ray, CT, and MRI before operation. **(L–Q)** x-ray and CT at 3 days after operation. **(R–U)** x-ray at 1 years follow-up. **(V–X)** x-ray and CT at 2.5 years follow-up.

**Figure 2:**
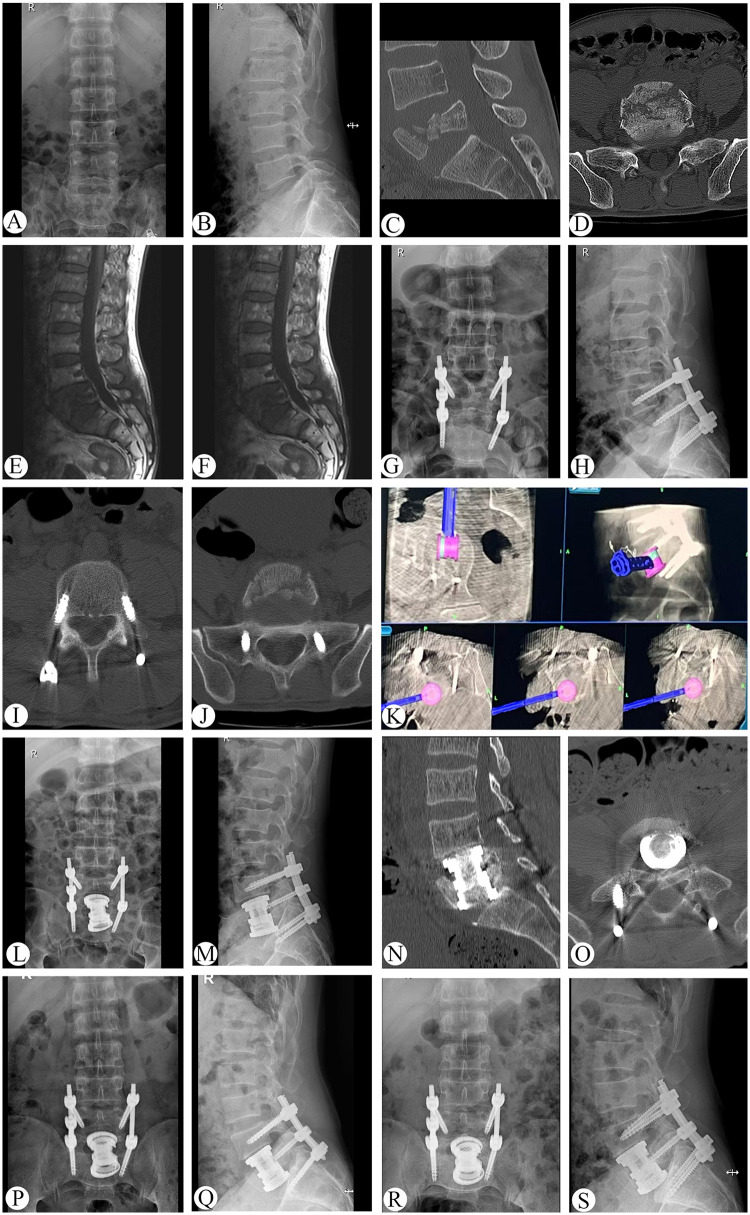
Preoperative, postoperative, and follow-up radiographs of a 21-year-old patient who had dated L5 vertebral fractures with kyphotic deformity managed by anterior vertebrectomy and reconstruction with O-arm navigation (case 15). **(A–F)** x-ray, CT, and MRI before operation. **(G–J)** x-ray and CT after posterior operation. **(K)** Real-time three-dimensional imaging of O-arm navigation. **(L–O)** x-ray and CT at 3 days after operation. (**P,Q)** x-ray at 1 years follow-up. **(R,S)** x-ray and CT at 2.7 years follow-up.

Table 2 summarizes the clinical efficacy and radiological evaluation of patients pre and postoperation. Representative cases are shown in Figure 1 (Case 8) and Figure 2 (Case 15).

**Table 2:** The clinical efficacy and radiological evaluation of patients pre and postoperation.

Variable	Pre-op	1w Post-op	3-month follow-up	Final follow-up	*F* value	*P* value
VAS	5.6 ± 1.1	3.5 ± 0.5	2.2 ± 0.6	1.0 ± 0.8	4.73	0.012
ODI	69.9 ± 3.2	32.0 ± 4.9	22.1 ± 5.0	11.8 ± 3.9	25.88	<0.001
LCA	37.8 ± 2.4°	12.4 ± 5.2°	12.2 ± 2.9°	14.4 ± 3.7°	8.46	<0.001
VAMHR	35.9 ± 5.6%	93.3 ± 4.9%	92.5 ± 2.7%	92.1 ± 2.1%	36.00	<0.001

LCA, local Cobb angle; VAMHR, vertebral anterior margin height ratio. Continuous variables were compared across the four time points using one-way repeated-measures ANOVA. Pairwise comparisons were performed using Bonferroni correction when the overall test was significant.

## 4 Discussion

### 4.1 Management of old L5 traumatic fractures with kyphotic deformity

Kyphotic deformities resulting from lumbar vertebral fractures pose significant challenges in surgical management, with various approaches offering distinct advantages and limitations. Posterior approaches, including pedicle subtraction osteotomy and posterior column resection, are widely used due to their familiarity and ability to address global spinal alignment (13–16). However, these techniques are less effective in decompressing anterior neural structures and may require extensive posterior instrumentation, increasing the risk of complications such as adjacent segment degeneration (17). Anterior approaches, such as anterior subtotal corpectomy and reconstruction, provide direct access to the anterior spinal column, allowing for effective correction of local kyphosis, neural decompression, and stability reconstruction (10, 18). However, anterior surgery can be technically demanding, with potential risks of vascular injury and prolonged operative time (6). Combined approaches attempt to leverage the strengths of both anterior and posterior techniques, offering superior deformity correction but at the cost of increased surgical time, complexity, and patient morbidity (19).

In this study, the improvement in radiological and clinical outcomes suggests that anterior vertebrectomy with reconstruction can allow direct ventral decompression, restore anterior column height, correct local kyphosis, and reconstruct load-bearing stability across the lumbosacral junction. An important finding of the present series is that correction was achieved early after surgery and was largely maintained during follow-up. The improvement in LCA and VAMHR, with only minimal loss of correction at the final follow-up, suggests that this approach provides not only immediate deformity correction but also sufficient structural support for medium-term alignment maintenance. This is clinically relevant at L5, where the lumbosacral junction is exposed to considerable axial and shear forces, and where loss of correction may compromise pain relief, function, and implant durability (1, 6, 10). The improvement in pain and disability should also be interpreted beyond the numerical changes in VAS and ODI. Neurological recovery in this series was significant overall but modest in magnitude, with most patients remaining unchanged and a subset improving by one ASIA grade. This pattern is not unexpected in chronic injuries, in which prolonged compression and post-traumatic remodeling may limit neurological reversibility. Nevertheless, the absence of neurological deterioration and the improvement observed in some patients suggest that direct anterior decompression may still offer meaningful benefit in selected cases. Accordingly, the goals of surgery in old L5 fractures should be understood as pain relief, functional improvement, prevention of further neurological deterioration.

### 4.2 Complication management and intraoperative precautions

The complex vascular architecture of the lumbosacral region, characterized by the left common iliac vein traversing obliquely across the spine anterior to the L5 vertebra, renders the anterior exposure among the most challenging surgical routes to execute. Similarly, vascular injuries sustained during surgery represent the most recurrent complications encountered in anterior spinal procedures, with reported incidences ranging from 7.9% to 13.8% (20, 21). Intraoperative considerations for anterior vertebrectomy and reconstruction include careful planning and precise execution to minimize complications and ensure surgical success. The use of O-arm navigation significantly enhances surgical precision, providing real-time three-dimensional imaging that facilitates accurate placement of pedicle screws and ensures safe resection of bony margins (9). This advanced navigation system reduces intraoperative errors and complications associated with traditional fluoroscopy, such as screw misplacement or incomplete decompression, making it a valuable tool for managing complex spinal deformities (22). Additionally, handling the iliolumbar veins requires meticulous attention, as these structures are at high risk of injury during anterior approaches. Understanding the detailed anatomy and variations of these veins is critical, and techniques such as preoperative imaging and careful dissection can reduce the risk of bleeding and vascular complications (23). In anatomically difficult cases, assistance from vascular or general surgeons may further improve the safety of exposure (20, 21, 23).

The resection of bony margins is another critical step, requiring precision to achieve neural decompression without causing excessive damage to adjacent structures. To securely position an expandable cage in the ventral space, it is imperative to meticulously dissect around the L4, L5, and S1 nerve roots, thereby fully exposing the dural sac. This maneuver eases pressure on neural components, creating a secure environment for cage placement. The most intricate phase involves excising the lowest segment of the L5 vertebral body and navigating near the axillary region of the L5 nerve root. In order to reduce bleeding and avoid affecting the blood supply to the spinal cord after surgery, the segmental vessels on the contralateral side are preserved, and the fractured vertebrae are not completely removed, but part of the cortical bone on the contralateral side is retained. Upon preparing the endplates of both L5 and S1 for cage integration, achieving a precise anatomical fit becomes paramount between the cage's terminal ends and the respective vertebral surfaces. Subsequent to cage expansion, upon attaining ideal sagittal and coronal alignments, the posterior section of the pedicle screw assembly can progress towards completion. These intraoperative strategies, combined with technological advancements like the O-arm system, allow for effective correction of kyphotic deformity, safe decompression, andreconstruction of anterior spinal column stability (23).

### 4.3 Limitations

This study has several limitations. First, it is a retrospective case series without a control group, which limits causal inference and precludes direct comparison with other surgical strategies or nonoperative treatment. As an inherent limitation of this design, selection bias and uncontrolled confounding cannot be excluded. Second, the sample size was relatively small, despite the rarity of this condition, which limits the robustness of subgroup analyses. Third, this was a single-center study, and all procedures were performed by experienced surgeons, which may reduce the generalizability of the findings. Finally, the follow-up period was moderate, and longer-term studies are needed to evaluate the durability of correction, fusion status, and implant-related outcomes.

## 5 Conclusion

In conclusion, anterior vertebrectomy combined with O-arm navigation presents a promising approach for managing old L5 traumatic vertebral fractures with kyphotic deformity. In this retrospective single-center series, the procedure was associated with significant improvement in pain, disability, neurologic function, vertebral height restoration, and sagittal alignment, with a high fusion rate and a low rate of major complications. Nevertheless, the findings should be interpreted in light of the study design, and further prospective multicenter comparative studies are needed.

## Data Availability

The original contributions presented in the study are included in the article/Supplementary Material, further inquiries can be directed to the corresponding author/s.

## References

[1] Meyer M Noudel R Farah K Graillon T Prost S Blondel B Isolated unstable burst fractures of the fifth lumbar vertebra: functional and radiological outcome after posterior stabilization with reconstruction of the anterior column: about 6 cases and literature review. Orthop Traumatol Surg Res. (2020) 106:1215–20. 10.1016/j.otsr.2020.03.014 32354682

[2] Bogdan L Galgano M. Technical nuances of a posterior-only L5 vertebrectomy with anterior column reconstruction. Surg Neurol Int. (2020) 11:325. 10.25259/SNI_473_2020 33194259 PMC7656019

[3] Butler JS Fitzpatrick P Ni Mhaolain AM Synnott K O'Byrne JM. The management and functional outcome of isolated burst fractures of the fifth lumbar vertebra. Spine. (2007) 32:443–7. 10.1097/01.brs.0000255076.45825.1e 17304135

[4] Seybold EA Sweeney CA Fredrickson BE Warhold LG Bernini PM. Functional outcome of low lumbar burst fractures. A multicenter review of operative and nonoperative treatment of L3-L5. Spine. (1999) 24:2154–61. 10.1097/00007632-199910150-00016 10543015

[5] Likhachev SV Zaretskov VV Arsenievich VB Ostrovskij VV Shchanitsyn IN Shulga AE Treatment tactics for patients with isolated injuries of the fifth lumbar vertebra. Sovrem Tekhnologii Med. (2021) 13:31–9. 10.17691/stm2021.13.5.04 35265347 PMC8858414

[6] Allain J. Anterior spine surgery in recent thoracolumbar fractures: an update. Orthop Traumatol Surg Res. (2011) 97:541–54. 10.1016/j.otsr.2011.06.003 21820378

[7] Zhu Y Zhu S Li Y Wang K. Robot-assisted, conventional fluoroscopy (C-arm), O-arm navigation, and freehand pedicle screw fixation in thoracolumbar spine fracture surgery: a network meta-analysis. Orthop Surg. (2025) 17(12):3302–17. 10.1111/os.70189 41074646 PMC12685484

[8] Delcont MR Ou-Yang DC Burger EL Patel VV Wessell NM Kleck CJ. Alternative uses of O-arm and stealth navigation technology over 10 years: the university of Colorado experience. Orthopedics. (2023) 46:e89–97. 10.3928/01477447-20220719-04 35876781

[9] Banat M Wach J Salemdawod A Bahna M Scorzin J Vatter H. The role of intraoperative image guidance systems (three-dimensional C-arm versus O-arm) in spinal surgery: results of a single-center study. World Neurosurg. (2021) 146:e817–21. 10.1016/j.wneu.2020.11.013 33181376

[10] D'Aquino D Tarawneh AM Hilis A Palliyil N Deogaonkar K Quraishi NA. Surgical approaches to L5 corpectomy: a systematic review. Eur Spine J. (2020) 29:3074–9. 10.1007/s00586-020-06617-y 33025193

[11] Elmose SF Andersen GO Carreon LY Sigmundsson FG Andersen MO. Radiological definitions of sagittal plane segmental instability in the degenerative lumbar spine - A systematic review. Global Spine J. (2023) 13(2):523–33. 10.1177/21925682221099854 35606897 PMC9972266

[12] Bridwell KH Lenke LG McEnery KW Baldus C Blanke K. Anterior fresh frozen structural allografts in the thoracic and lumbar spine. Do they work if combined with posterior fusion and instrumentation in adult patients with kyphosis or anterior column defects? Spine. (1995) 20:1410–8. 10.1097/00007632-199506020-00014 7676341

[13] Elnady B Shawky A Abdelrahman H Elmorshidy E El-Meshtawy M Said GZ. Posterior only approach for fifth lumbar corpectomy: indications and technical notes. Int Orthop. (2017) 41:2535–41. 10.1007/s00264-017-3570-7 28733847

[14] Cavagnaro MJ Tavolaro C Orenday-Barraza JM Farhardi D Baaj AA Bransford R. Burst fractures of the fifth lumbar vertebra: case series and systematic review. J Clin Neurosci. (2022) 103:163–71. 10.1016/j.jocn.2022.07.017 35907351

[15] Pham MH Tuchman A Chen TC Acosta FL Hsieh PC Liu JC. Transpedicular corpectomy and cage placement in the treatment of traumatic lumbar burst fractures. Clin Spine Surg. (2017) 30:360–6. 10.1097/BSD.0000000000000312 28937458

[16] Lin B Chen ZW Guo ZM Liu H Yi ZK. Anterior approach versus posterior approach with subtotal corpectomy, decompression, and reconstruction of spine in the treatment of thoracolumbar burst fractures: a prospective randomized controlled study. J Spinal Disord Tech. (2021) 25:309–17. 10.1097/BSD.0b013e3182204c53 21637134

[17] Zeng Y Chen Z Sun C Li W Qi Q Guo Z Posterior surgical correction of posttraumatic kyphosis of the thoracolumbar segment. J Spinal Disord Tech. (2013) 26:37–41. 10.1097/BSD.0b013e318231d6a3 21964450

[18] Yao Y Yan J Jiang F Zhang S Qiu J. Comparison of anterior and posterior decompressions in treatment of traumatic thoracolumbar spinal fractures complicated with spinal cord injury. Med Sci Monit. (2020) 26:e927284. 10.12659/MSM.927284 33211674 PMC7684844

[19] El-Sharkawi MM Koptan WM El-Miligui YH Said GZ. Comparison between pedicle subtraction osteotomy and anterior corpectomy and plating for correcting post-traumatic kyphosis: a multicenter study. Eur Spine J. (2011) 20:1434–40. 10.1007/s00586-011-1720-y 21336510 PMC3175905

[20] Shousha M El-Saghir H Boehm H. Corpectomy of the fifth lumbar vertebra, a challenging procedure. J Spinal Disord Tech. (2014) 27:347–51. 10.1097/BSD.0b013e318260dced 22688613

[21] Silvestre C Mac-Thiong JM Hilmi R Roussouly P. Complications and morbidities of Mini-open anterior retroperitoneal lumbar interbody fusion: oblique lumbar interbody fusion in 179 patients. Asian Spine J. (2012) 6:89–97. 10.4184/asj.2012.6.2.89 22708012 PMC3372554

[22] Yang P Chen K Zhang K Sun J Yang H Mao H. Percutaneous short-segment pedicle instrumentation assisted with O-arm navigation in the treatment of thoracolumbar burst fractures. J Orthop Translat. (2019) 21:1–7. 10.1016/j.jot.2019.11.002 32042590 PMC6997617

[23] Davis M Jenkins S Bordes S Iwanaga J Loukas M Uribe J Iliolumbar vein: anatomy and surgical importance during lateral transpsoas and oblique approaches to lumbar spine. World Neurosurg. (2019) 128:e768–72. 10.1016/j.wneu.2019.04.252 31077904

